# Primary Cardiac Angiosarcoma in a Young Male: Rare Presentation With Right Atrial Endocardial Dissection and Right Coronary Artery Tumor Fistula

**DOI:** 10.1002/ccr3.72760

**Published:** 2026-05-29

**Authors:** Nehzat Akiash, Ramtin Rezaee Kalantari, Somayeh Abbaspour, Mahlasadat Masoumi, Narges Eslami, Nasibeh Akiash, Mahboubeh Pazoki, Shahab Rajabzadeh, Soroush Mostafavi

**Affiliations:** ^1^ Atherosclerosis Research Center Ahvaz Jundishapur University of Medical Sciences Ahvaz Iran; ^2^ Department of Cardiac Surgery, Atherosclerosis Research Center Ahvaz Jundishapur University of Medical Sciences Ahvaz Iran; ^3^ Clinical Research Development Unit Ahvaz Jundishapur University of Medical Sciences Ahvaz Iran; ^4^ Golestan Hospital Ahvaz Jundishapur University of Medical Sciences Ahvaz Iran; ^5^ Department of Cardiology, School of Medicine Hazrat‐e Rasool General Hospital, Iran University of Medical Sciences (IUMS) Tehran Iran

**Keywords:** cardiac angiosarcoma, cardiac surgery, endocardial dissection, hemopericardium, tumor fistula

## Abstract

We report the case of a 19‐year‐old man who presented with several months of exertional dyspnea and recent‐onset hemostasis. Initial imaging revealed multiple pulmonary nodules. Days later, he was referred to a specialized center with chest pain and signs of pericarditis. Transthoracic echocardiography (TTE) showed pericardial effusion with hematoma, while transesophageal echocardiography (TEE) revealed a vascularized mass in the right atrial wall supplied by the right coronary artery (RCA), with blood leakage into the pericardial space. Due to concern for cardiac perforation, emergency surgery was decided. Intraoperatively, coronary angiography was performed prior to chest closure due to the anatomical complexity of the RCA. Pathology and immunohistochemistry analysis of cardiac, pericardium, and lung specimens confirmed the diagnosis of primary cardiac angiosarcoma. This case represents the aggressive behavior, diagnostic challenges, and therapeutic complexity of cardiac angiosarcoma, and highlights the importance of early diagnosis, accurate imaging, and a multidisciplinary approach.

## Key Clinical Message

Doppler TEE provides critical diagnostic value in detecting coronary fistulization, directly influencing surgical strategy. Tumor‐related coronary distortion significantly increases intraoperative risk. Early multimodality imaging, particularly in unstable patients, enables timely diagnosis and coordination. Integrated preoperative assessment and multidisciplinary management are essential to optimize surgical planning and improve clinical outcomes.

## 1 Introduction

Angiosarcoma is the most common primary malignant tumor of the heart, occurring predominantly in the right atrium and adjacent to the atrioventricular groove [1]. It arises from vascular endothelial cells and is known to have a high propensity for metastasis and local recurrence [2]. The presence of hemorrhagic and irregular masses in the right atrium, even in the absence of other evidence of malignancy, strongly suggests primary cardiac angiosarcoma [3]. The prognosis of this disease is poor due to its anatomical location and metastatic potential, and it is often associated with hemorrhagic pericardial or pleural effusions [4, 5]. Angiosarcoma tends to invade the myocardial wall, cardiac cavities, valves, vascular structures, and pericardium [6]. The most common clinical manifestations vary depending on the tumor's location, size, and extent of tumor invasion. Symptoms such as dyspnea, chest pain, and palpitation often arise due to complications including superior vena cava obstruction, cardiac tamponade, congestive heart failure, and arrhythmias. The right‐sided form usually presents as a large, infiltrative mass with nonspecific symptoms, whereas the left‐sided form is more commonly associated with dyspnea due to local obstruction and heart failure [7].

## 2 Case Presentation

The patient, a 19‐year‐old male, presented to a medical center with several months of exertional dyspnea accompanied by recurrent hemoptysis over the preceding 3 weeks. He denied systemic symptoms such as weight loss, fever, or night sweats. Initial chest computed tomography (CT) scan showed multiple pulmonary nodules and pericardial effusion, but no further diagnostic workup was performed at that time.

One week later, the patient was referred to our emergency department with progressive dyspnea and chest pain consistent with a pericarditis pattern. TTE showed pericardial effusion with hematoma in the pericardial space with hemodynamic findings consistent with pre‐tamponade. Also, a suspicious thickening of the lateral wall of the right atrium was seen, leading to the decision to perform TEE.

### 2.1 Paraclinical Findings

**FIGURE 1:**
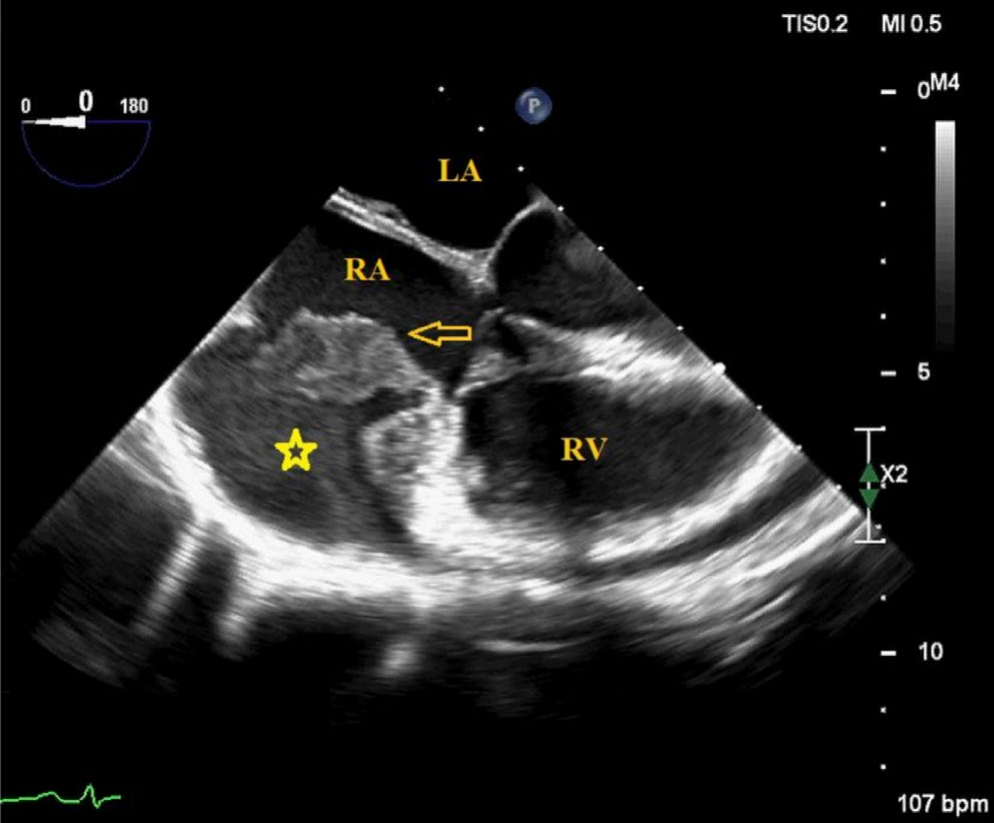
TEE mid‐esophageal view (0°) showed right atrial endocardial dissection, blood dissemination in the subendocardial space (asterisk), presence of a mass on the right atrial endocardium (arrow). LA, Left Atrium; RA, Right Atrium; RV, Right Ventricle.

**FIGURE 2:**
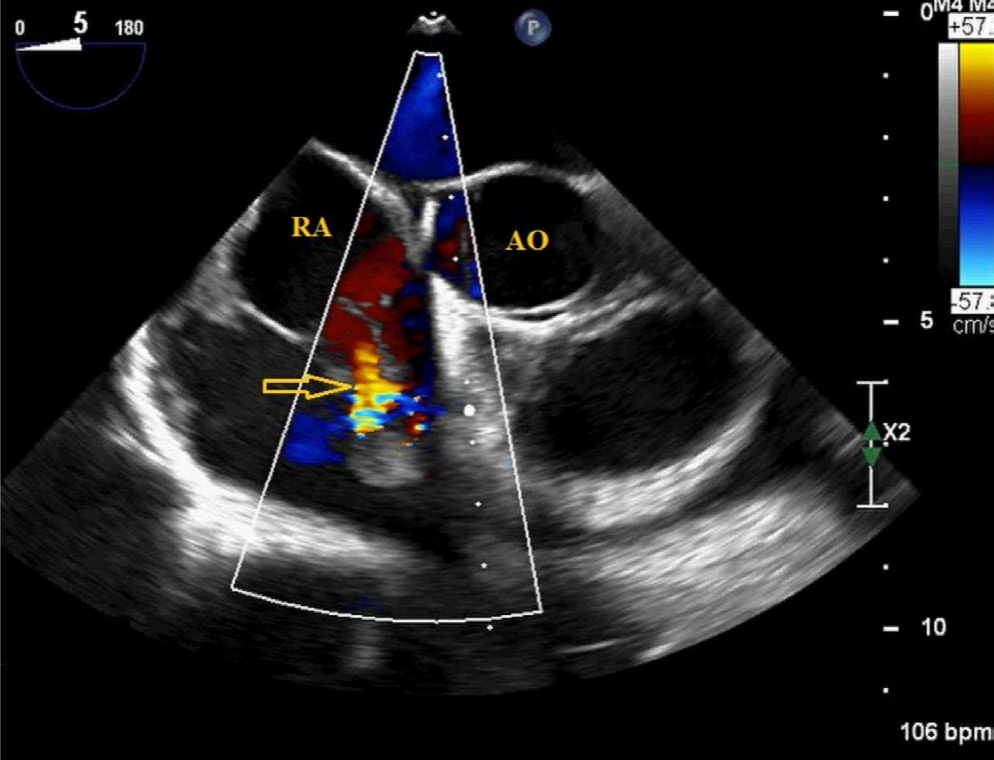
TEE mid‐esophageal view (5°) showed a vascularized mass in the right atrial wall with a fistulous connection from the RCA into the tumor (arrow). RA, Right Atrium; AO, Aorta.

TEE revealed a right atrial endocardial mass (Figure 1) with a prominent vascular supply originating from the right coronary artery (RCA) which also demonstrates a fistulous communication into the tumor (Figure 2, Video 2). Additionally, there was evidence of right atrial endocardial dissection (Figure 1 and Video 1), with blood dissemination into the subendocardial space which lies immediately beneath the endocardium (Figures 1 and 2, Videos 1 and 2), and a hematoma in the pericardial space, suggesting perforation of the RA wall—including myocardium and epicardium—into the pericardial cavity (Video 3).

**VIDEO 1:** TEE mid‐esophageal view (0°) shows right atrial endocardial dissection, blood dissemination in the subendocardial space, presence of a mass on the right atrial endocardium (arrow). LA, Left Atrium; RA, Right Atrium; RV, Right Ventricle. Video content can be viewed at https://onlinelibrary.wiley.com/doi/10.1002/ccr3.72760.

**VIDEO 2:** TEE mid‐esophageal view (5°) shows a vascularized mass in the right atrial wall with a fistulous connection from the RCA into the tumor (asterisk). LA, Left Atrium; RA, Right Atrium; RV, Right Ventricle. Video content can be viewed at https://onlinelibrary.wiley.com/doi/10.1002/ccr3.72760.

**VIDEO 3:** TEE mid‐esophageal view (105°) showed hematoma in the pericardial space (arrows), suggesting perforation of the RA wall into the pericardium. Video content can be viewed at https://onlinelibrary.wiley.com/doi/10.1002/ccr3.72760.

**FIGURE 3:**
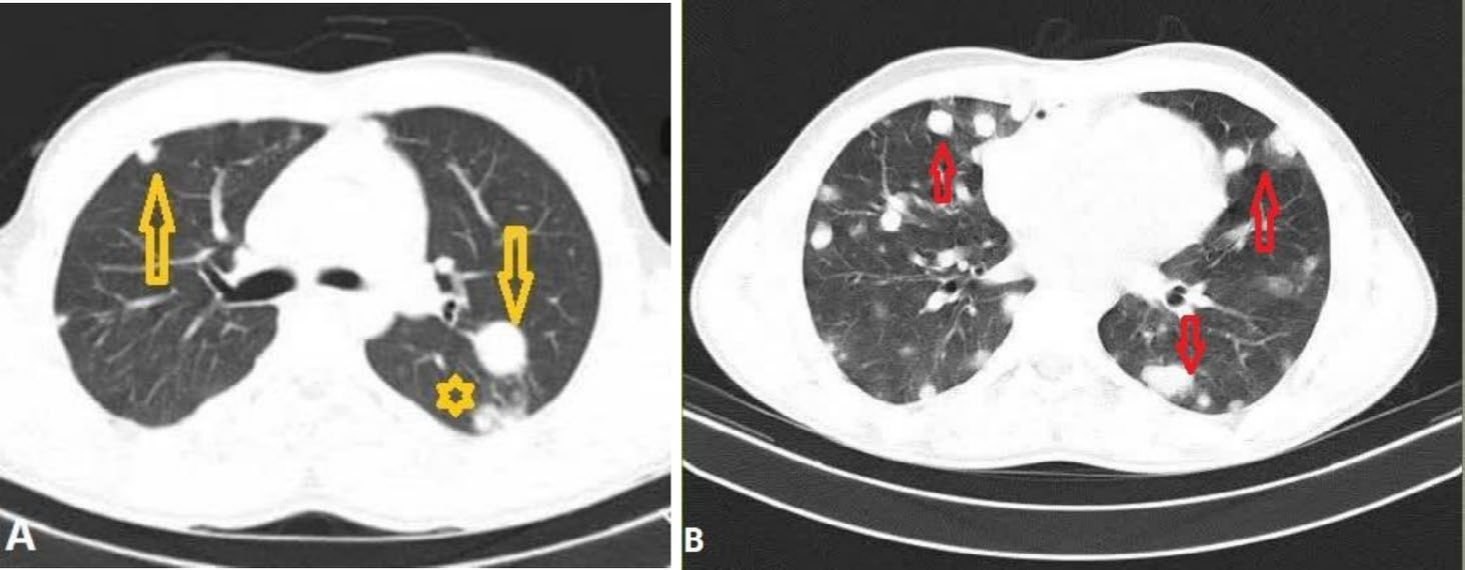
Preoperative contrast‐enhanced chest CT in the lung window demonstrates bilateral solid nodular lesions (yellow arrows) accompanied with surrounding ground‐glass opacity (asterisk) (A). A post‐treatment contrast‐enhanced chest CT obtained 10 months after the initiation of chemotherapy shows multiple pulmonary metastases (red arrows) (B).

A contrast‐enhanced chest CT (CCT) and a contrast‐enhanced abdominal CT scan were obtained immediately preoperatively following TEE to exclude any other origin of the cardiac mass, such as mediastinal or abdominopelvic tumors. Contrast‐enhanced CCT revealed multiple nodules (Figure 3A) in the lung parenchyma and umbilical lymphadenopathy. Abdominal ultrasound, testicular ultrasound, and contrast‐enhanced abdominal CT scan were unremarkable.

### 2.2 Therapeutic Intervention

Given the imaging findings and the possibility of cardiac perforation, the decision was made to perform emergency surgery. Consent was obtained as chest pain and severe dyspnea worsened, and the patient was transferred to the operating room.

The cardiac surgery was performed with separate cannulation of the inferior vena cava (IVC) and superior vena cava (SVC), and a total cardiopulmonary bypass (CBP) was established. Following the induction of cardiac arrest, the RA was exposed. The tricuspid valve (TV) and RV wall were assessed and appeared grossly intact, with no evidence of tumor invasion. All mass lesions within the RA were successfully resected. Subsequently, the large RA wall defect was repaired using a pericardial patch after total resection of the tumor. Additionally, the fistula between the RCA and the mass was excised. Finally, a saphenous vein graft (SVG) was anastomosed to the RCA distal to the resected segment of its proximal portion. During surgery, the team encountered technical challenges, including the small caliber of the distal part of RCA and difficulty identifying its course beyond the fistula connection. Concerns regarding the graft placement on the coronary venous system and the presence of severe right ventricular (RV) dysfunction prompted coronary angiography prior to final chest closure. After confirming the correct position of the graft on RCA, the chest was closed (Videos 4 and 5).

**VIDEO 4:** Coronary angiography shows a cut segment of the proximal part of RCA following fistula ligation. RCA, Right Coronary Artery. Video content can be viewed at https://onlinelibrary.wiley.com/doi/10.1002/ccr3.72760.

**VIDEO 5:** Coronary angiography reveals SVC of RCA. SVG, Saphenous Vein Graft; RCA, Right Coronary Artery. Video content can be viewed at https://onlinelibrary.wiley.com/doi/10.1002/ccr3.72760.

### 2.3 Post‐Surgical Follow‐Up

**FIGURE 4:**
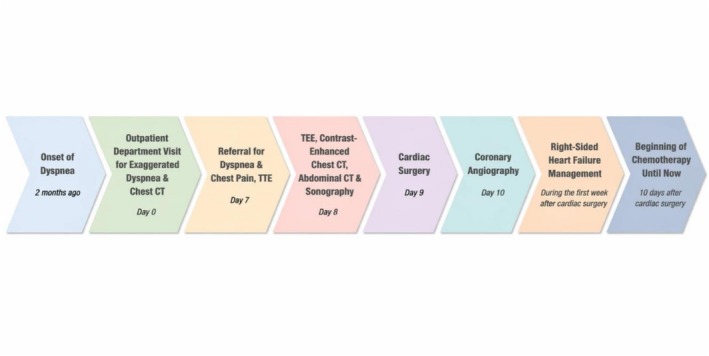
Timeline of the patient's clinical course, including clinical events, diagnostic investigations, surgical treatment, and postoperative management.

During the postoperative recovery period, the patient showed signs of severe RV failure and marked inflammation in the RV wall. Based on the new echocardiographic findings, the patient was transferred to the heart failure service and underwent specialized treatment by the heart failure fellowship team for 1 week. By the end of the recovery period, a marked improvement in RV function was observed, and he was discharged in stable condition. The patient was subsequently referred to the oncology department for further management. During follow‐up at the cancer center, cardiac evaluation revealed no evidence of cardiac mass regrowth, significant RV dysfunction, or significant pericardial effusion. However, the pulmonary metastatic burden increased (Figure 3B), accompanied by a deterioration in the patient's overall clinical condition during ongoing chemotherapy. The patient initially received Adriamycin, Ifosfamide, and Mesna (AIM regimen) as first‐line therapy. In response to the progression of pulmonary metastases, Docetaxel was subsequently added to the therapeutic regimen. The patient's clinical course, including symptom onset, diagnostic evaluations, surgical management, and subsequent treatments, is summarized in the timeline (Figure 4).

### 2.4 Pathological Findings

**FIGURE 5:**
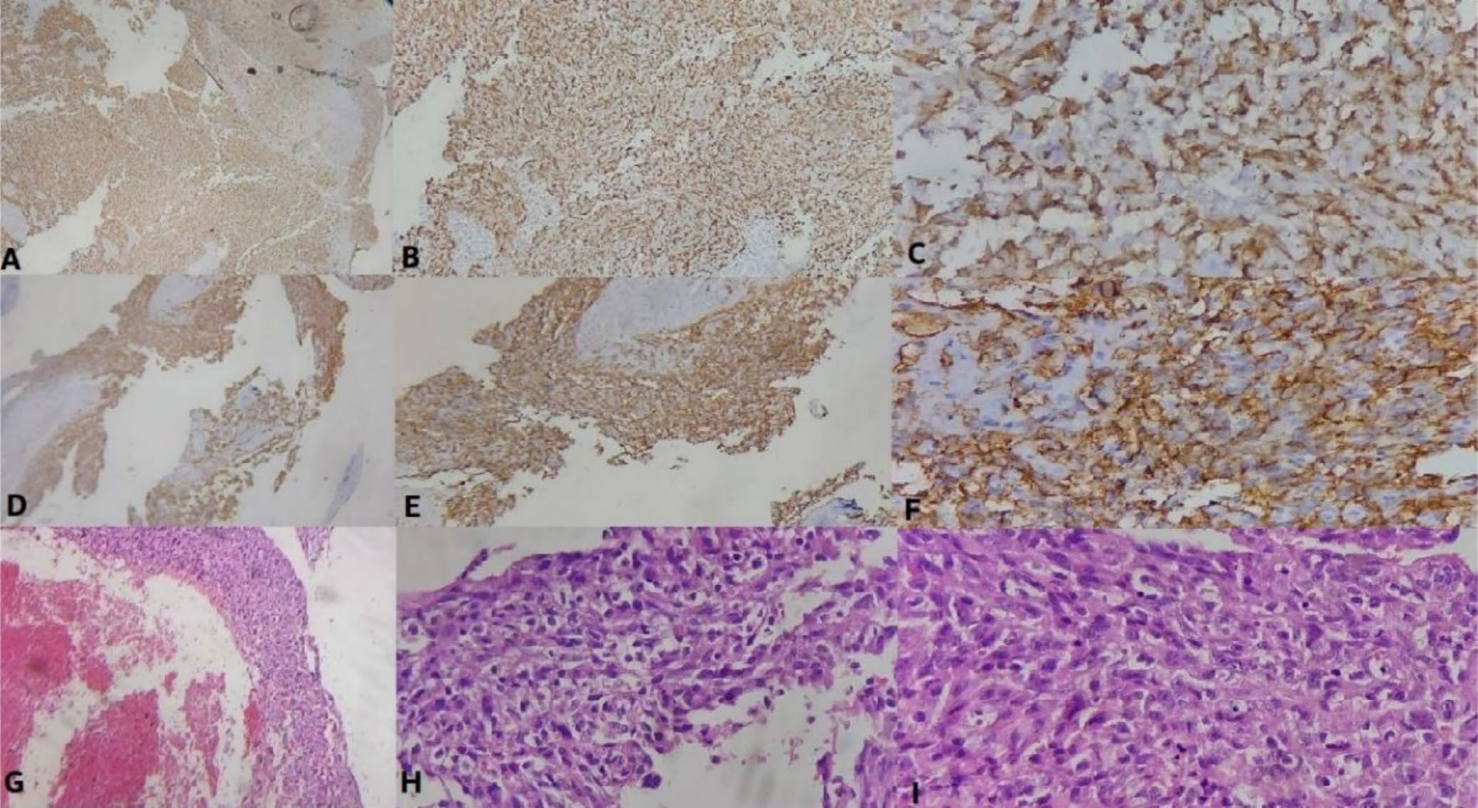
CD34 (A, B, and C, 4×, 10×, and 40×, respectively) and CD31 (D, E, and F, 4×, 10×, and 40×, respectively) Immunostaining of cardiac angiosarcoma show strong expression of vascular marker. Histopathological sections (G, H, and I, 4×, 10×, and 40×, respectively) show numerous mitotic figures indicating high proliferative and aggressive tumor activity.

Tissue samples taken from the right atrial mass, pericardial space, and pulmonary nodules were sent for pathology and immunohistochemistry (IHC) examination. The final results confirmed the diagnosis: primary cardiac angiosarcoma (Figure 5).

## 3 Discussion

Primary cardiac angiosarcoma is a high‐grade, aggressive malignancy originating from the endothelial cells of blood vessels [8]. Angiosarcoma typically presents between the ages of 30 and 50 years. Studies indicate that the incidence in males is approximately twice that in females. The underlying cause of this sex disparity remains uncertain, although hormonal imbalance has been suggested as a possible contributing factor. Right‐sided angiosarcoma tends to be bulky, more infiltrative, and highly aggressive, in contrast to left‐sided lesions, which are less bulky and have a lower metastatic potential [9]. Angiosarcoma often presents with nonspecific symptoms such as dyspnea, chest pain, and signs of right‐sided heart failure. Serious complications, including cardiac rupture and tamponade, are indicative of the tumor's aggressive nature and its invasion of the adjacent cardiac structures [10].

Metastases commonly present at the time of diagnosis of angiosarcoma. The lungs are the most frequent site of involvement. Secondary metastasis may also affect the liver, spleen, lymph nodes, adrenal glands, and bones [11].

Echocardiography is the primary and cost‐effective method for diagnosing cardiac masses, while TEE provides more detailed information about the mass characteristics [12]. In our case, fistula formation of the RCA in cardiac angiosarcoma led to dissection of the endocardial layer of the RA wall and blood flow entering the subendocardial space, representing a rare presentation. Kui Tang et al. reported a case of primary cardiac angiosarcoma complicated by spontaneous rupture of the RA wall and RCA, resulting in localized massive pericardial effusion. Color Doppler demonstrated flow into the pericardial cavity through the disrupted RA wall and perforated RCA [13]. In a case report published in 1999, the patient presented with wall thickening of a dilated RA. Doppler analysis demonstrated elevated flow velocity and turbulence within the RA cavity, and angiography suggested an RCA–RA fistula (Qp/Qs = 1.5) with associated neovascularization [14]. In another reported case, pericardiocentesis was performed for bloody cardiac tamponade. Following subsequent hemodynamic deterioration, TTE demonstrated re‐accumulation of the pericardial effusion. Angiography revealed contrast extravasation from the proximal RCA into the RA and pericardial space. Emergent surgical exploration ultimately identified a lesion invading the RCA and establishing a fistulous communication with the pericardial sac [15].

Advanced imaging, such as CT and CMR, provides comprehensive information about the location, extent, and characteristics of the surrounding tissue. CMR is particularly effective in differentiating between benign and malignant lesions [16].

In primary cardiac angiosarcoma, MRI often reveals heterogeneous signal intensity and hyperintense hemorrhagic foci. Central liquefaction necrosis and pericardial enhancement are frequently observed [17].

Despite the cellular heterogeneity in AS, immunohistochemical markers such as CD31, CD34, ERG, and factor VIII are used in conjunction with clinical findings to confirm the endothelial origin of the tumor [11]. The treatment of choice for AS is complete surgical resection of the tumor. Therefore, early diagnosis and a multidisciplinary approach are keys to improving patient prognosis [18]. Any detected cardiac mass should be evaluated surgically with an intraoperative frozen section and histopathological confirmation. Endomyocardial and transbronchial biopsies are rarely diagnostic for angiosarcoma. The treatment strategy depends on tumor location, local invasion, and distant metastasis [9].

The median overall survival of cardiac angiosarcoma is approximately 12–14 months [8]. In early‐stage cardiac angiosarcoma, immediate surgical resection is recommended [18], as was done in the reported patient. Anthracyclines are generally considered for the treatment of angiosarcomas; however, their use may be limited due to potential cardiotoxicity [19]. Adjuvant therapies, including chemotherapy and radiotherapy, have been demonstrated in studies to enhance local control of tumors and diminish recurrence rates. Therefore, incorporating such therapies into the treatment plan for these patients should be a key consideration in clinical practice [20].

## 4 Conclusion

Preoperative surgical planning supported by a thorough diagnostic assessment is essential to reducing intraoperative challenges and optimizing surgical outcomes. In this case, Doppler TEE proved valuable in identifying coronary fistulization, which significantly influenced diagnostic assessment and surgical planning. Tumor‐related distortion of the coronary artery further increased surgical risk, highlighting the need for meticulous preoperative evaluation. Prompt recognition of these features through multimodality imaging and coordinated multidisciplinary management is essential to optimize surgical strategy and improve clinical outcomes. Additionally, monitoring the patient's condition and administering appropriate treatment based on pathological findings—along with the measures adopted in follow‐up visits‐ contribute significantly to achieving optimal clinical outcomes and mitigating potential complications throughout the treatment process.

## Author Contributions

**Nehzat Akiash:** conceptualization, data curation, supervision, writing – original draft. **Ramtin Rezaee Kalantari:** data curation, visualization, writing – original draft. **Somayeh Abbaspour:** conceptualization, writing – original draft. **Mahlasadat Masoumi:** data curation, visualization, writing – original draft. **Narges Eslami:** validation, writing – original draft. **Nasibeh Akiash:** validation, visualization, writing – original draft. **Mahboubeh Pazoki:** supervision, visualization, writing – review and editing. **Shahab Rajabzadeh:** writing – original draft, writing – review and editing. **Soroush Mostafavi:** conceptualization, writing – review and editing.

## Funding

The authors have nothing to report.

## Consent

Written consent had been obtained from the patient and is available on request from the authors.

## Data Availability

Data available on request from the authors.
